# Systems of Care in Cardiogenic Shock

**DOI:** 10.3389/fcvm.2021.712594

**Published:** 2021-09-16

**Authors:** Miguel Alvarez Villela, Rachel Clark, Preethi William, Daniel B. Sims, Ulrich P. Jorde

**Affiliations:** ^1^Division of Cardiology, Department of Medicine, Montefiore Medical Center, Albert Einstein College of Medicine, New York, NY, United States; ^2^Division of Cardiology, Jacobi Medical Center, Albert Einstein College of Medicine, New York, NY, United States; ^3^Division of Cardiology, Banner University Medical Center, Tucson, University of Arizona, Tucson, AZ, United States

**Keywords:** cardiogenic shock, systems of care, AMI-CS, AHF-CS, shock team, hub and spoke

## Abstract

Outcomes for cardiogenic shock (CS) patients remain relatively poor despite significant advancements in primary percutaneous coronary interventions (PCI) and temporary circulatory support (TCS) technologies. Mortality from CS shows great disparities that seem to reflect large variations in access to care and physician practice patterns. Recent reports of different models to standardize care in CS have shown considerable potential at improving outcomes. The creation of regional, integrated, 3-tiered systems, would facilitate standardized interventions and equitable access to care. Multidisciplinary CS teams at Level I centers would direct care in a hub-and-spoke model through jointly developed protocols and real-time shared decision making. Levels II and III centers would provide early access to life-saving therapies and safe transfer to designated hub centers. In regions with large geographical distances, the implementation of telemedicine-cardiac intensive care unit (CICU) care can be an important resource for the creation of effective systems of care.

## Introduction

Cardiogenic shock (CS) is a life-threatening condition that begins with an initial insult leading to hypoperfusion and can progress to multiorgan failure and death. Effective treatment requires early recognition and time-sensitive interventions to restore perfusion. Despite the widespread adoption of primary percutaneous coronary interventions (PCI) and the technological advancements made in temporary circulatory support (TCS), mortality for CS patients has remained largely unabated over the last decade (1). Today, 30-day mortality for CS due to any etiology is close to 40–47% in clinical trials and 30–51% in registry studies (2).

Large disparities in outcomes exist, however, across different care environments. In the United States (US), CS patients treated in large, urban and left ventricular assist device (LVAD)-capable centers, have the lowest mortality rates (3, 4) while those in smaller, rural hospitals have the highest mortality rates, along with the lowest rates of early angiography, PCI and access to TCS (5). Notably, current data shows that nearly half of patients with acute myocardial infarction and cardiogenic shock (AMI-CS) are being treated in low volume centers (6).

Recently published studies have demonstrated that initiatives to standardize care for CS patients within integrated care systems can lead to large improvements in clinical outcomes (7, 8). These models, akin to those adopted in other time-sensitive conditions, can facilitate efficient access to different tiers of care for patients with different severities of CS, which could improve the existing disparities. Herein, we describe the current state of systems of care in CS and propose what an ideal system might look like.

## Current Landscape

CS remains the main cause of death among patients with acute myocardial infarction (AMI), and now complicates close to 10% of cases (9). Currently, inpatient mortality for AMI-CS is estimated between 31 and 41%. Patients are increasingly presenting with higher clinical complexity, older age, greater comorbid burden and more complex culprit lesions (6). Higher overall use of primary PCI has not sustainably decreased mortality (10), but shorter times between first medical contact and PCI do seem to improve survival in AMI-CS patients (11). In the US, the number of PCI-capable centers has grown at a faster rate than the population growth, but these centers are unequally distributed, ranging between per capita 3–4/1 to 12/1 million. Distance between centers also varies greatly, reaching as far as 150 miles in some regions (12).

Between 2011 and 2013, PCI centers classified as suburban and rural performed 49% of all PCIs for AMI-CS in the US. Private and community hospitals performed 90% of these PCIs, while tertiary care centers performed only 10%. Data from the National Cardiovascular Data Registry indicates that in-hospital mortality as a whole is rising for this patient population, regardless of the treating center's characteristics (6). However, other large registry-based analyses suggest there may be higher survival for AMI-CS patients treated in large, urban or LVAD-capable centers (3, 6, 13).

Acute heart failure CS (AHF-CS), is increasingly recognized as a common etiology for CS, now accounting for 30–50% of cases depending on the hospital setting (14, 15). Reported mortality for these patients varies widely depending on the data source, likely reflecting important disparities in care. In a recent report from the Cardiogenic Shock Working Group, a research consortium of large academic centers, in-hospital mortality for AHF-CS was 26% (13), while in a contemporary analysis of the National Inpatient Sample (NIS) including all hospital types, mortality was 48% (15).

Access to TCS and physician patterns of device use also show wide variations. In a recent survey study of cardiac surgery centers, the IABP was offered in 92% of centers, followed by the Impella (Abiomed, Danvers, MA) in 78% and VA-ECMO in 66%. This survey also indicates that nearly one-third of physicians consider TCS before PCI in AMI-CS patients, and two-thirds do so after PCI (5). Patients admitted to larger hospitals (≥600 beds) are more likely to receive TCS than those admitted to smaller ones ( ≤ 200 beds) (16). Accordingly, in the United States, over 80% of VA-ECMO cases are performed in large, urban, teaching institutions (17). Use of MCS is also lower in patients older than 65 (15), women, African Americans, non-privately insured patients, and patients with low-income status (18).

Disparities in outcomes for CS patients seem to reflect these differences in management. In the US, patients in the Midwest and West have significantly lower in-hospital mortality than those in the Northeast, where lower rates of primary PCI and TCS are also noted. Meanwhile, patients in the South have the highest mortality of any region (4, 19). Patients admitted to urban and larger hospitals, with higher resource availability, have better outcomes than those in rural and smaller hospitals (4). Although in-hospital mortality is higher amongst Hispanics (74%) and African Americans (65%), these differences disappear when controlled for access to primary PCI (20). Similarly, the higher mortality observed amongst women with AMI-CS (21), is reduced with the use of standardized management algorithms (22).

## Developing Systems of Care in CS

The above data support the notion that creating multi-tier systems that allow for timely and equal access to standardized care for patients with CS, would improve outcomes. As an initial step, an effort has been made in recent years to identify CS “centers of excellence” that could serve as hubs to receive patients within a larger conglomerate of hospitals.

According to an American Heart Association scientific statement on CS management, designated CS hospitals should have access to a critical care unit, 24/7 PCI capability, support from cardiac surgery and access to TCS including VA-ECMO as well as durable LVADs (2). Notably, in a survey from 2019, only 40% of the 6,000 responding centers have access to all TCS support modalities, 20% report access to PCI only, 16% do not have Cardiac Surgery programs, and 6% do not have 24/7 access to PCI (5). This highlights the current existence of different tiers of care for CS patients. A regional system should be designed to stabilize patients in lower-level centers and provide timely transfer to larger center with access to higher care for those who are most severely ill (5, 23).

### Barriers

Several key issues should be considered in the creation of effective systems of care for CS (Table 1). First, timely diagnosis and identification of patients is problematic. Currently, there is no universally accepted definition of CS, as none seem to effectively identify all cases (2). Using hard cut-points in objective clinical and laboratory parameters is limited by the variety of presentations seen with the different etiologies. A highly sensitive definition can identify more patients earlier, but depending on the adopted model of care, it could also result in the unnecessarily frequent mobilization of large amounts of resources. The definition of the CS stages by the Society of Cardiovascular Angiography and Interventions (SCAI) has provided a classification of shock that contemplates pre-shock stages that can help in early diagnosis (24). A recent study using machine learning technology was able to further stratify patient risk by identifying three distinct CS phenotypes upon presentation: “Non-congested,” “Cardiorenal,” and “Cardiometabolic” among patients with both, AMI-CS and AHF-CS (25). These classifications represent important steps toward guiding early therapeutic interventions.

**Table 1:** Current Barriers to the creation of effective regional systems of care for CS.

**Current barriers**	**Potential solutions**
No standard definition of CS	Convened definition developed within the region and accepted by all three hospital levels
Gaps in standard of care for CS	Jointly developed regional management protocol with distinct pathways for AMI-CS and AHF-CS
Long geographical distances between spokes and hub	Three-tier system with CCL serving as base for initiation of monitoring and care in Level II centers Development of tele-CCU system to guide care from Level I centers
No definition for CS centers of excellence	Development of a three-tiered system supported by national or international professional societies

*AHF-CS, acute heart failure—cardiogenic shock; AMI-CS, acute myocardial infarction—cardiogenic shock; CCL, cardiac catheterization lab; CCU, coronary care unit; CS, cardiogenic shock*.

Second, important inconsistencies exist in current guidelines addressing the management of CS patients. For instance, early revascularization in AMI-CS is the only therapeutic intervention that receives a class I indication in both ACC/AHA and ESC guidelines. Meanwhile the use of a pulmonary artery catheter receives no grade in the ACC/AHA STEMI guidelines, but a class I recommendation in American heart failure guidelines and a class IIb grading in European guidelines. Larger discrepancies are seen in the recommendations for IABP use. The American guidelines give IABP a class IIa recommendation in STEMI, and European guidelines consider it a Class III indication in STEMI and HF guidelines. The use of other TCS devices is graded as class IIb by ESC guidelines and ACC/AHA STEMI guidelines, but a class IIa in the American HF guidelines. These discrepancies reflect the low level of evidence underpinning most of these recommendations, as well as differences in publication timing (2013 for ACC/AHA STEMI and HF guidelines vs. 2016 and 2017 for ESC HF and STEMI guidelines) (26).

Moreover, the majority of existing clinical trials in CS were done in AMI-CS patients. However, depending on the hospital setting, AHF-CS is potentially as frequent as AMI (27–29). Patients with AHF-CS have a distinct clinical phenotype and also respond differently to TCS than AMI-CS patients do, and often present to all hospital types (30, 31). Hence, a multi-tiered system needs to develop shared management algorithms with distinct pathways for these different patient phenotypes.

Third, geographical distance can have a negative impact in certain regions. A careful balance is needed between the institution of early therapeutic interventions and the transfer of patients to higher level of care centers where therapy can be escalated. For example, short first-medical-contact to balloon times in AMI-CS (11) should be prioritized, but protocolized institution of hemodynamic support should also occur as early as possible in selected patients. Lower-level hospitals should have streamlined access to designated teams in larger centers who can aid with early management decisions, coordinate transfer and deploy to these smaller centers as needed. Emergency medical services available in the region will obviously play an important role in this effort.

Finally, an established definition for “CS centers of excellence” is needed to help with the appropriate identification of hub centers within a region. Clear identification of these centers would not only help standardize access to care, but could also garner strong support from governing bodies to facilitate issues like sharing of physician credentialing across hospitals and state lines, and sharing of costs between transferring and receiving centers.

## Current Initiatives

### Hub and Spoke Model

The Hub-and-Spoke model is based on the current model for STEMI, trauma and stroke referral systems (23). The original hub-and-spoke model for CS was implemented in New York for patients for treatment of post-cardiotomy CS. Each spoke site was within 250 miles of the hub. The hub center was contacted when a patient was in refractory shock for over 12 h following surgery. This model was successful in increasing the survival rate by 66% (2).

Hospitals within such networks are organized into 3 levels: Level I centers act as dedicated shock hubs with access to advanced TCS, cardiothoracic surgery, durable LVAD, hypothermia protocols and a robust multidisciplinary team culture in place. They accept transfers from both level II and level III hospitals, which differ in their ability to perform PCI and institute IABP or Impella support. Level II and III hospitals need to have protocols in place for out of hospital cardiac arrest (OHCA) and advanced cardiac life support (ACLS), as well as the ability to rapidly identify and transfer CS patients safely. Emergency Department staff in levels II and III centers should have access to bedside transthoracic echo (TTE) (7). Published data from formally established models in Spain and in the Mayo Clinic in Arizona showed increased survival rates using this approach (14, 31). Distances between spokes and hub centers can be a limitation in certain areas like the rural United States, where immediate transfer may require a large amount of resources (14, 32).

The hub and spoke model effectively centralizes the management of the most complex patients in level I centers with higher use of TCS and higher volumes of CS (2). Studies performed in patients receiving ECMO (33), PCI, CABG (2), and LVADs (3) have all demonstrated better outcomes in the facilities with the highest volume. This model can hence serve to concentrate scarce resources in a given region.

The Cardiac-RESCUE trial identified that only one sixth of the 1,000 ICU beds in the Paris region were able to provide ECMO support. Their mobile hub shock team quadrupled the number of ICU beds able to provide this therapy (34) and served as a solution to the geographic disparities. A similar mobile team was used in a network of hospitals in Spain (14) where a shock team could travel to level II and III hospitals to evaluate patients and provide MCS as needed. They eventually transferred 42% of patients to their level I center, improving survival to discharge from 51 to 64%. Arizona had similar outcomes, with 25 of 27 patients transferred, 55% of which had MCS placed prior to transfer (35). Key to the hub and spoke model's success is the close collaboration between the hub and the spoke sites to develop joint protocols and provide training for the effective implementation of these protocols at each site (36).

Based on experiences from the development of STEMI systems, the ability for facilities to carry this out depends on geography (rural vs. urban), regional resources, and state lines. The transition to a hub and spoke model can be complicated by misaligned existing referral patterns and lack of funding and supplies for mobile teams (2). The development of a national CS database will be important to developing regionalized care guidelines and improving outcomes (2).

### Cardiogenic Shock Protocols

The Detroit Cardiogenic Shock Initiative (DCSI) evaluated the use of early MCS in patients with AMI-CS that were undergoing PCI. Four Detroit hospitals participated with adherence to the protocol which included: early PCI with invasive assessment of HD and use of Impella based on established criteria, aim for TIMI III flow with use of vasodilators as needed, and post intervention assessment of invasive hemodynamics with escalation or de-escalation in support as needed. Forty-one patients were included in the initial study, with 85% surviving until device removal, and 76% surviving until discharge (37). The DCSI has been expanded to become the National Cardiogenic Shock Initiative (NCSI) with over 80 institutions adopting the initial DCSI protocol by December 2020. Preliminary data has been made available on their latest report, which included 406 patients enrolled at 80 sites with a 71% survival to discharge between 2016 and 2020 (38).

Consistent with prior data emphasizing the importance of early interventions in AMI-CS patients, the DCSI found that with every 60 min delay to MCS, there was a 9.9% increase in mortality (7, 37). Their protocol enabled early identification of patients with a documented plan of action, which drastically improved time to MCS (85 ± 63 min) without significantly delaying revascularization. This is an example of how the incorporation of shock protocols into regionalized care systems has the potential to uniformly improve outcomes.

### Shock Teams

Based off of the success seen with team-based care for STEMIs, in-hospital cardiac arrest and rapid response teams, some hospitals have developed CS teams (7). These generally consist of physicians with backgrounds in Critical Care, Interventional Cardiology, Heart Failure, and Cardiothoracic Surgery. The team is activated with a single phone call when a patient with CS is identified (31, 39, 40) This multidisciplinary team can assess the patient at the bedside or through chart review, and make decisions on different therapeutic interventions. Some shock teams are mobile and can move to the referring hospital for support, while other models stay in the hospital and coordinate urgent transfers to their center. After initial stabilization or clarification of the goals of care, involvement of the shock team can be de-escalated (41).

The University of Utah Shock Team approach, the INOVA team-based care model, the Canadian shock team and the French Cardiac-RESCUE study, all used versions of shock teams for the identification and treatment of patients with CS. These studies often blend the use of a shock team with a hub-and-spoke model as described above. In the Utah experience, 67.5% of patients were transferred to the hub hospital (31% of them after TCS institution at the referring hospital) (39). Fifty-two percent were transferred to the tertiary center in the INOVA experience, 74% in the Canadian shock team publication (29) and 84% were successfully transferred to the main VA-ECMO centers in the Cardiac-RESCUE study (34).

At the University of Utah, the shock team decreased in-hospital and 30-day mortality from 61 to 48% between 2015 and 2018 (39). Patients with post-cardiotomy shock were not included in this initiative. The Utah CS team was activated using criteria defined as “CS suspected by the treating physician.” Activation occurred via a 24/7 on-call heart failure specialist who would initially assess the case and then coordinate and organize the team's response. A protocolized early escalation to TCS was favored for patients who remained hypoperfused and refractory to medical therapy. Notably, TCS device type did not predict survival and involvement of the CS team did not delay the time to institution of TCS. This initiative has been sustainable, and the shock team remained active 4 years after its creation (39).

The INOVA model consists of a multidisciplinary shock team in which all members are contacted simultaneously via a single phone call after CS is identified using simple clinical parameters (hypotension, hypoperfusion, elevated lactate). Specific pathways are defined for patients with AMI-CS and patients with AHF-CS. Their management strategy has five key areas of focus: early identification of CS, early universal right heart catheterization (RHC) to guide tailored treatment, early TCS institution, limiting inotropic and vasopressor use, and patient recovery and survival. A continued improvement in survival was seen with this approach as survival rates increased from 47% in 2016, to 58% in 2017, and 77% in 2018 (42). This system combines the use of a basic protocol with a shock team and a hub-and-spoke model with over half of patients transferred to their tertiary care center from smaller hospitals (40).

CS teams highlight the value of the simultaneous bedside assessment by specialists from different disciplines in improving management decisions. But, given the wide variations in access and practice patterns mentioned above, these models are not feasible for study in a RCT setting. CS teams are also highly resource intensive. They require the creation and maintenance of an on-call team as well as a parallel track for 24/7 activation of the cardiac catheterization laboratory (41). With less stringent activation criteria, a high incidence of false calls and inappropriate use of resources can lead to increased costs and compromise the program's sustainability. This effect has been studied previously in STEMI systems (43). A tiered activation model, where cases are first filtered through an on-call intensivist or HF specialist as seen in the Utah experience after hours and with the Canadian shock team during all activations, could limit resource exhaustion (29, 39). This did not increase time to TCS in the Utah experience, but it was not directly measured in the Canadian report (29).

## Discussion

### Integration of Hub-and-Spoke Models, Protocols, and CS Teams: What Should the System of Care Look Like?

The ideal system of care for CS would integrate elements from all three models described above (Table 2). Within a region, hospitals would be aligned within a hub-and-spoke model. Care at the spoke sites would be guided by established protocols and supported by a CS team at the hub center.

**Table 2:** Basic characteristics of a cardiogenic shock protocol.

Jointly developed by collaboration between hubs-and-spokes within a region
Adapted for each region's resources and characteristics
Distinct pathways for AMI-CS and AHF-CS
Provides guidance on appropriate initial testing and hemodynamic monitoring
Provides guidance for care at each tier of the system
Provides guidelines for triage and safe transfer of patients

*AHF-CS, acute heart failure—cardiogenic shock; AMI-CS, acute myocardial infarction—cardiogenic shock*.

A uniform definition of CS would be shared across all three levels of care in the system. In our opinion this definition should be sensitive enough to identify early cases but should also be able to discriminate between patients in different risk groups. The early recognition of CS in our institution is based on the early identification of the following data:

1. Patient's risk of CS: Does the patient have an acute or recent MI? Does the patient have known cardiomyopathy?
2. Is the patient exhibiting signs of hypoperfusion, congestion and/or hypotension? Cool skin, pulmonary edema, peripheral edema or altered mental status regardless of systemic BP?
3. Does the patient have laboratory evidence of end-organ dysfunction such as new or worsening renal failure, elevated transaminases or elevated lactic acid?

**Figure 1:**
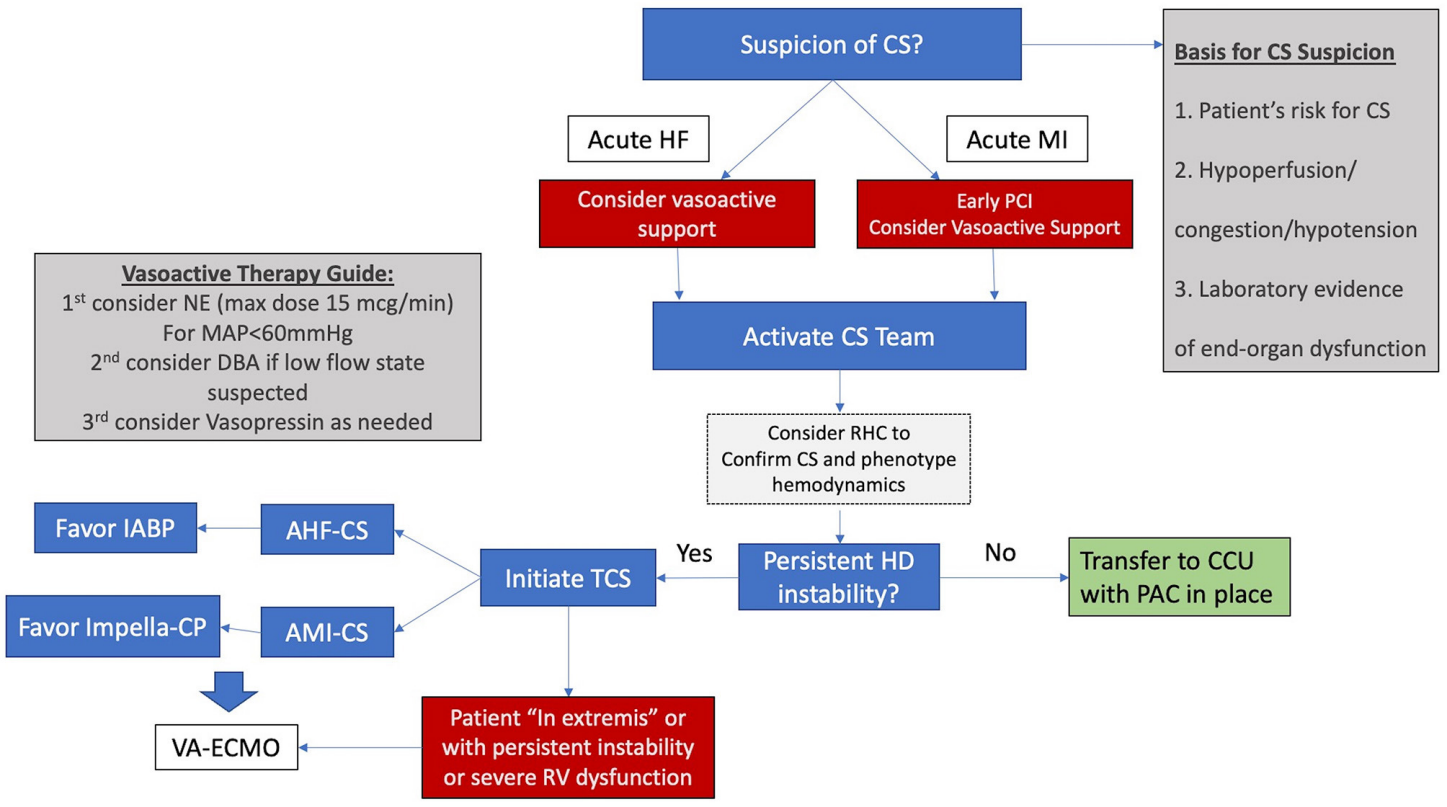
Simplified algorithm for initial management for CS. AHF-CS, acute heart failure cardiogenic shock; AMI-CS, acute myocardial infarction cardiogenic shock; CCU, coronary care unit; CS, cardiogenic shock; DBA, dobutamine, HD hemodynamic; HF, heart failure; IABP, intra-aortic balloon pump; MAP, mean arterial pressure; NE, norepinephrine; PAC, pulmonary artery catheter; RHC, right heart catheterization; RV, right ventricle; STEMI, ST elevation myocardial infarction; TCS, temporary circulatory support; VA-ECMO, veno-arterial extracorporeal membrane oxygenation.

Using these three points, patients can be identified either in the emergency department or in the CCL. After diagnosis, the algorithm in Figure 1 should be followed. At our institution the HF attending on service in the CCU serves as the first point of contact for calls regarding CS patients. The HF attending collects relevant initial data and recommends initial steps in treatment. The surgical and critical care teams are then activated as needed.

Our preferred initial vasopressor is norepinephrine and care is taken to avoid doses above 15 mcg/min. Vasopressin is our next vasopressor of choice, usually at a dose of 0.04 mcg/kg/min. Dobutamine and Milrinone are used as inotropes and an early RHC is encouraged either in the CCU or the CCL.

For patients with AHF-CS our initial TCS of choice is the IABP and for patients with AMI-CS, our initial device of choice is the Impella CP. When hemodynamic instability persists or severe RV failure is present, our choice is commonly to proceed with VA-ECMO in order to restore end-organ perfusion and prevent further deterioration. VA-ECMO in our institution is implanted by the cardiac surgery team who is deployed to smaller hospitals within our system for implant and transfer of unstable patients to our main hospital in the “Moses Campus.” This hospital houses our cardiac transplantation and LVAD program and serves as the hub within our referral network.

To become a hub within a CS system, a hospital would ideally obtain accreditation as a “Level I” center through a certification process sponsored by a professional society or a pertinent governing body. This process would ensure that these centers have the necessary resources for this role, including 24/7 PCI capability and dedicated CCU care, access to all modalities of TCS including VA-ECMO, cardiac surgery support, a multi-disciplinary cardiogenic shock team and access to advanced cardiac therapies like LVAD and transplant (Table 3).

**Table 3:** Basic characteristics of a hub cardiogenic shock team.

Multidisciplinary: Interventional cardiology, cardiac intensive care, cardiac surgery, heart failure
Guides care at a system level by proposing protocols and aiding in organization of resources
Provides ongoing education to staff at the spoke level
Available for consultation 24/7 via single phone call
Has a mobile unit capable of deploying from Level I to Levels II and III centers

**Figure 2:**
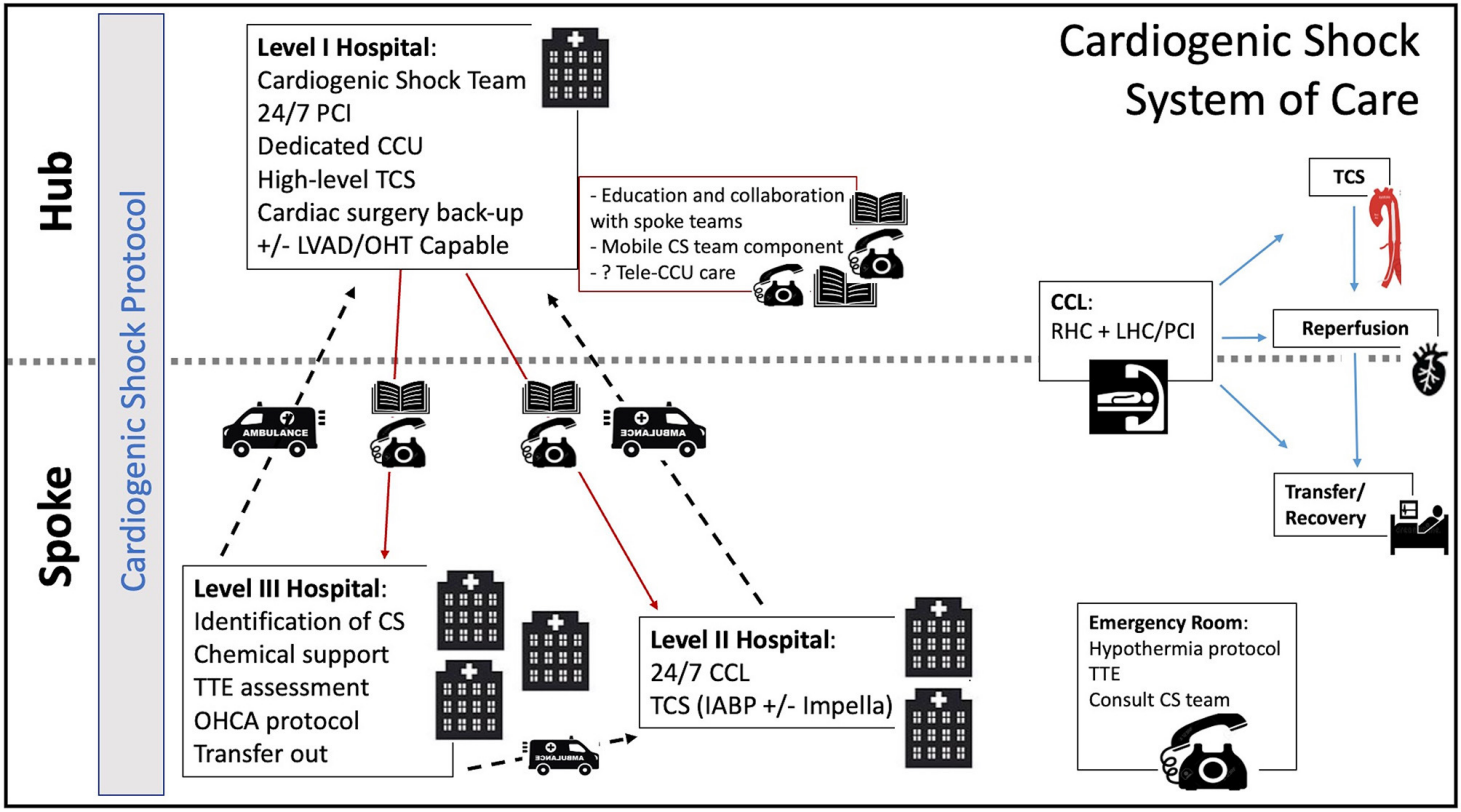
Cardiogenic Shock System of Care. CCU, coronary care unit; CCL, cardiac catheterization lab; CS, cardiogenic shock; IABP, intra-aortic balloon pump; LVAD, left ventricular assist device; OHCA, out of hospital cardiac arrest; OHT, orthotropic heart transplant; PCI, percutaneous coronary intervention; RHC, right heart catheterization; TCS, temporary circulatory support; TTE, transthoracic echocardiogram.

Through the same process, spoke hospitals would obtain accreditation as Level II and Level III centers. This 3-tiered system, similar to what is seen in trauma care, has been previously proposed by some authors (2, 7, 32). In this model, a Level III center would identify patients in or at risk of CS and triage them to a Level II or I center within the region depending on the patient's needs. Level II centers, more widely available than Level I centers, would have 24/7 PCI capability. At this level, the cardiac catheterization laboratory (CCL) would serve as the base for initial interventions, including early angiography and PCI for AMI-CS patients, but also RHC and TCS institution for patients with all etiologies of CS (Figure 2).

The availability of MCS at spoke centers is rapidly changing. Although the IABP is currently the most widely available TCS modality across would-be Level II centers, the Impella is gaining wide availability in certain regions. The advent of newer technologies like the LifeSparc system (TandemLife, Pittsburgh) could also facilitate the more widespread access to VA-ECMO in a CCL setting without the need for an on-site perfusionist or cardiac surgery support. Patients supported with higher level TCS would be immediately transferred to the Level I center.

Level II and III centers would operate based on a clear algorithm focused on best practices developed with their designated hub center. Clear pathways would be provided for patients with AMI-CS and AHF-CS. This CS protocol would also include guidance on early institution of hemodynamic monitoring, preferred initial pharmacologic support, and the early identification of patients needing escalation to TCS. This protocol would be coupled with an intensive training and awareness campaign for the early identification of CS patients in all hospital departments. In addition, the emergency department would be able to perform rapid and reliable bedside TTE assessment. Our institutional protocol for the early management of CS is outlined as an example in Figure 1.

At the designated Level I center, a CS team would be available via a single phone call on a 24/7 basis to provide early consultation and assist in shared decision making. Access to the spoke hospital's electronic health records could help the CS team have direct access to the patient's primary data. In areas where centers are spread over large geographical distances and immediate transfers may not be feasible, an intensive care telemedicine model could be adopted. This model has shown promise in adult critical care, reducing mortality and improving adherence to best practices (40). A robust telemedicine model could eventually offload the need for beds at the hub center, allowing for ongoing care of appropriately selected patients at Level II centers. Such telemedicine systems can be financially sustainable if compensation models are properly aligned (44). As greater capacity develops in Level II and III hospitals, patients could be transferred back to these centers to alleviate the demand for beds in the hub centers.

Ideally, a component of the hub's CS team should be mobile, able to deploy to the spoke sites to aid in management and institute higher levels of TCS not primarily available at the local level. As mentioned above, mobile teams have been successful in improving access to care and reducing mortality in France and Spain (14, 34).

## Conclusions

The current landscape in CS is characterized by a persistently high mortality along with important variations in access to care and physician practice patterns. While most of the data guiding CS care comes from studies performed in AMI-CS, the relative incidence of AHF-CS is growing. A universal definition for CS remains elusive and important gaps in knowledge limit the adoption of standards of care. The implementation of hub-and spokes models, CS protocols, and CS teams have all shown promising results at improving access to high-level care and improved short-term survival. The creation of accredited 3-tiered hospital systems within defined geographical regions can serve to direct care through ongoing education, the development of protocols, and shared patient management through a centralized multi-disciplinary CS team.

## Author Contributions

MA: contributed to design of paper and wrote first draft. RC: assisted in writing first draft, organizing references, and designed the figure. PW, DS, and UJ: reviewed manuscript and provided editorial comments, adding additional references, and editing figures and tables. All authors contributed to manuscript revision, read, and approved the submitted version.

## Conflict of Interest

The authors declare that the research was conducted in the absence of any commercial or financial relationships that could be construed as a potential conflict of interest.

## Publisher's Note

All claims expressed in this article are solely those of the authors and do not necessarily represent those of their affiliated organizations, or those of the publisher, the editors and the reviewers. Any product that may be evaluated in this article, or claim that may be made by its manufacturer, is not guaranteed or endorsed by the publisher.

## Abbreviations

ACLS: advanced cardiovascular life support
AHF: acute heart failure
AHF-CS: acute heart failure cardiogenic shock
AMI: acute myocardial infarction
AMI-CS: acute myocardial infarction—cardiogenic shock
CICU: cardiac intensive care unit
CS: cardiogenic shock
DSI: Detroit shock initiative
HD: hemodynamics
IABP: intra-aortic balloon pump
LVAD: left ventricular assist device
MCS: mechanical circulatory support
NCSI: National cardiogenic shock initiative
OHCA: out of hospital cardiac arrest
PCI: percutaneous coronary intervention
STEMI: ST elevation myocardial infarction
TCS: temporary circulatory support
TTE: transthoracic echocardiogram
US: United States
VA-ECMO: venoarterial—extracorporeal membrane oxygenation.
